# A lightweight LBM detection transformer with multi-scale feature fusion for citrus-picking robots

**DOI:** 10.3389/fpls.2026.1847836

**Published:** 2026-06-15

**Authors:** Ke Gao, Baijing Wu, Jiren Gu, Guanghui Yan

**Affiliations:** 1 School of Electronic and Information Engineering, Lanzhou Jiaotong University, Lanzhou, China; 2 School of Engineering, Jiujiang Polytechnic University of Science and Technology, Jiujiang, China

**Keywords:** citrus recognition, cross-scale feature fusion, harvesting robots, RT-DETR, smart agriculture, vision system

## Abstract

To address false positives and missed detections caused by complex orchard backgrounds and small target regions during citrus harvesting, this study proposes a lightweight LBM Detection Transformer for citrus recognition in citrus-picking robots, integrating cross-scale feature fusion. Firstly, a large-kernel attention module (LKAM) is introduced for feature extraction, where multi-scale large-kernel convolutions are employed to comprehensively capture global, local, and texture information while maintaining the lightweight nature of the model. Secondly, a multi-scale linear attention (MSLA) Transformer encoder is incorporated to further enhance feature discrimination, thereby enabling the network to focus more accurately on citrus targets and effectively suppress background interference. Finally, a bidirectional feature pyramid neck (BFPN) is designed to achieve efficient cross-layer feature fusion by integrating deep and shallow representations, which compensates for target information loss caused by occlusion. Experimental results on citrus orchard scenes demonstrate that, compared with the baseline model, the proposed LBM Detection Transformer reduces the parameter count by 5.54 M while improving mAP by 4.69%, mmAP by 5.14%, and R by 3.66%. These results indicate that the proposed method provides an accurate and lightweight solution for intelligent citrus management and automated harvesting applications.

## 1 Introduction

Jiangxi Province, China, is located in the subtropical humid monsoon climate zone and is recognized as one of the country’s major fruit-producing regions, where citrus products, particularly navel oranges, play a prominent role. In 2021, the citrus planting area in Jiangxi reached 20 million mu, ranking first nationwide, while navel orange production reached 17 million tons, ranking second in China. To promote high-quality agricultural development, Jiangxi Province issued the Opinions on Promoting High-Quality Development of Agriculture and Rural Areas and Striving to Build a Model Region for Rural Revitalization in the New Era, which explicitly identifies the fruit industry as one of the six key sectors targeted to achieve a trillion-yuan output value and further outlines a three-stage roadmap for rural revitalization. Although the total output value of China’s navel orange and citrus industry has exhibited a steady upward trend, net profit growth has remained relatively stagnant. This disparity primarily results from the high labor costs associated with citrus harvesting, which still relies heavily on manual labor and material inputs. Such labor-intensive practices are not only costly but also difficult to sustain in terms of efficiency, and they are prone to missing optimal harvesting windows, thereby adversely affecting citrus quality. Consequently, promoting the deployment and automation upgrading of navel orange and citrus harvesting robots is of critical importance for the sustainable development of the industry (Lu et al., 2024). By leveraging efficient visual recognition systems, harvesting robots can achieve accurate and robust citrus detection and picking, thereby significantly improving operational efficiency, reducing dependence on manual labor, and enabling effective identification of diseased fruits to ensure overall product quality. However, in complex orchard environments typical of navel orange and citrus production areas, robotic vision systems still face substantial challenges, including cluttered backgrounds, uneven illumination, and frequent occlusion among branches, leaves, and fruits. These factors often lead to degraded recognition accuracy and limited system stability. Therefore, developing a stable and accurate visual perception system for harvesting robots that can efficiently recognize citrus targets and reliably distinguish defective fruits constitutes a key technological prerequisite for achieving automated harvesting and effective quality control.

The research and widespread application of fruit harvesting robots constitute an essential component of future smart agriculture. According to the Made in China 2025 initiative, intelligent agricultural machinery and equipment have been identified as key development areas for advancing modern agriculture in China (Tang et al., 2020). Harvesting robots are capable of perceiving fruit-picking tasks in dynamic and unstructured agricultural environments, thereby enabling accurate fruit perception and recognition under complex conditions. With reliable visual perception, robots can acquire precise picking locations and subsequently control robotic systems to execute appropriate harvesting actions. At present, research on harvesting robots remains primarily at the stage of engineering development and demonstration. Although significant progress has been achieved in several core technologies, and related systems are gradually transitioning from laboratory environments to field-level validation and performance optimization, large-scale industrial deployment still faces substantial challenges, including the instability of visual perception systems, high implementation costs, and limited adaptability to diverse operational scenarios (Wang et al., 2024; Elaraby et al., 2022; Gu and Syafrudin, 2025). In the field of smart agriculture, (Chen et al., 2024b) reviewed the research progress of key technologies for fruit and vegetable harvesting robots, including visual sensing systems, target detection, localization, and end-effector design. Their study systematically analyzed existing limitations in these critical components and provided a comprehensive outlook on future development trends. (Zhang et al., 2024b) proposed a contact force calculation model for apple harvesting grippers based on force-closure theory, which effectively reduced fruit damage and insufficient grasping force during harvesting. To meet the demand for intelligent and efficient citrus harvesting in complex environments, (Xiao et al., 2024c) developed an optimized citrus recognition algorithm using YOLOv7 and designed a corresponding end-effector for citrus picking, thereby avoiding fruit damage during harvesting. Furthermore, (Zhang et al., 2024a) addressed the complexity of harvesting paths in mango picking operations by proposing a dynamic path planning algorithm, which optimized robotic harvesting routes and significantly improved harvesting efficiency. Current research on harvesting robots remains largely at the engineering demonstration stage. Existing visual systems generally rely on deep and complex networks to integrate features across different layers, resulting in high computational costs and limited real-time performance. Consequently, they struggle to simultaneously achieve robust recognition under occlusions, disease symptoms, and other disturbances in complex orchard environments while enabling lightweight deployment.

Among the various components of harvesting robots, the visual perception system represents one of the most critical core technologies (R M et al., 2025). However, in real-world dynamic orchard harvesting scenarios, navel orange and citrus harvesting robots are frequently confronted with dense foliage, adjacent fruits, and cluttered backgrounds, which often lead to severe target occlusion and incomplete visual information of citrus objects. Moreover, visual systems are required to identify whether harvesting targets exhibit quality-related defects, such as discoloration, melanosis, black spots, or canker, which directly affect the quality of navel oranges and citrus fruits (Chen et al., 2024a; Xue and Wang, 2025). These complex conditions impose higher requirements on the anti-interference capability, robustness, and real-time performance of recognition algorithms. With respect to citrus recognition algorithms, (Hu et al., 2024) proposed a citrus recognition method integrating random sampling and grid-based filtering strategies, thereby effectively addressing fruit occlusion and overlap challenges in complex orchard environments. (Zhou et al., 2024) introduced the YOLOv5 NMM (yolov5 with navel orange measure model), which significantly improved detection accuracy for navel oranges at different maturity stages and under occlusion and dense distribution by incorporating an additional small-object detection layer, attention mechanisms, and an optimized feature fusion network. (Zhao et al., 2025) focused on non-destructive and accurate discrimination of multiple navel orange varieties at different growth stages and proposed a dual-branch multimodal feature fusion convolutional neural network that integrates near-infrared hyperspectral imaging (HSI) with machine vision. By jointly analyzing internal fruit composition and external color–texture features, their method achieved accurate and reliable maturity assessment. To address challenges such as illumination variation, leaf occlusion, and the extremely small size of Huanglongbing-infected leaves in citrus disease detection, (Xiao et al., 2024a) proposed the YOLO-EAF (yolo-efficient asymptotic fusion) model. By introducing an efficient multi-scale attention module and adaptive spatial feature fusion, the model effectively enhanced fine-grained disease feature extraction and multi-scale fusion capability, thereby providing a new solution for intelligent orchard disease monitoring. Furthermore, techniques such as 3D object detection (Hou et al., 2024), stereo vision (Xiao et al., 2024b), monocular depth estimation–based methods (Ye et al., 2025), and semantic segmentation–based localization (Xie et al., 2024) have been introduced to alleviate missed detections caused by fruit occlusion. However, existing citrus disease detection methods generally rely on deep convolutional or Transformer architectures to perform complex feature interactions, resulting in large numbers of parameters and substantial computational redundancy, which hinders real-time inference on embedded platforms. Moreover, densely stacked network structures tend to suffer from feature attenuation and missed detections when handling occluded fruits, small disease spots, and complex background interference, making it difficult to simultaneously achieve high accuracy and lightweight deployment.

In summary, the navel orange and citrus industry occupies a pivotal position in Chinese agriculture, yet its high-quality development has long been constrained by the high and unstable costs of manual harvesting. The development of efficient and intelligent harvesting robots has therefore become an urgent need for industrial upgrading. However, in complex orchard environments, the stability and accuracy of robotic visual recognition systems remain challenged by occlusion, disease symptoms, and variable lighting conditions. To address these issues, this study proposes an LBM Detection Transformer for citrus target detection, which incorporates structural adaptation and lightweight modifications tailored to citrus harvesting tasks. Three key modules are introduced: LKAM (Large-Kernel Attention Module), BFPN (Bidirectional Feature Pyramid Network), and MSLA (Multi-Scale Linear Attention). Without relying on any pretrained weights, the model achieves accurate real-time detection of small occluded citrus targets under complex backgrounds. Specifically, LKAM enhances the perception of occluded small targets and suppresses background interference via multi-scale large-kernel convolutions; the MSLA Transformer encoder employs multi-scale linear attention to focus on targets and reduce false negatives and false positives; and BFPN adaptively fuses shallow and deep features to compensate for occlusion-induced feature attenuation, thereby improving detection robustness. The main contributions are summarized as follows:

1. LKAM. A large-kernel attention–based feature extraction strategy is proposed to enhance citrus target perception in complex orchard environments. Through a multi-scale large-kernel convolutional design, global, local, and texture features are comprehensively captured, thereby reducing interference from cluttered backgrounds and occlusion. This module strengthens the network’s ability to model long-range dependencies and improves the representation of small and partially occluded citrus targets.
2. MSLA transformer encoder. A multi scale linear attention transformer encoder is introduced to further enhance deep feature representations by leveraging convolutions with different receptive fields. This design enables the network to more accurately focus on citrus targets while effectively suppressing background interference, thereby significantly alleviating missed detections and false alarms under complex orchard conditions.
3. BFPN. A Bidirectional Feature Pyramid Network is designed to address feature attenuation of occluded targets during feature propagation. By jointly integrating shallow and deep features through adaptive weighted interactions, BFPN effectively and coherently combines fine grained spatial details with high level semantic information, thereby compensating for citrus target features lost due to occlusion and improving detection robustness.
4. Experimental results obtained from real-world citrus planting scenarios demonstrate that the proposed LBM Detection Transformer achieves lightweight and efficient citrus target detection performance. Compared with the baseline model, the parameter count is reduced by 5.54 M, while mAP, mmAP, and R are simultaneously improved by 4.69%, 5.14%, and 3.66%, respectively. These results indicate that the proposed method provides a stable and practical technical solution for intelligent citrus harvesting robots.

## 2 Dataset collection procedure and characteristics analysis

### 2.1 Overview of the study area

To evaluate the effectiveness of the proposed LBM Detection Transformer in citrus fruit disease detection scenarios, two datasets were selected: the publicly available OFDD (Faisal et al., 2023) (Orange Fruit Diseases Dataset) and a self-collected dataset from Jiangxi, JXDF (Citrus Fruit Diseases in Jiangxi). The OFDD dataset contains 700 images of citrus fruits covering five categories: Black Spot, Canker, Greening, Healthy, and Scab. Each diseased region in the images was annotated using CVAT with bounding boxes and subsequently converted into the YOLO format, enabling direct use for object detection tasks. The dataset integrates images from Mendeley Data and Kaggle, which were manually cleaned and reorganized. It preserves variations in lighting conditions, image quality, and disease progression from early to late stages, thereby enhancing the model’s generalization capability in real-world orchard environments.

The JXDF dataset constructed in this study comes from the real production scenes and some web crawler image data sets in Ganzhou City, Jiangxi Province, which is the largest navel orange and citrus cultivation region in China, thereby ensuring strong representativeness and authenticity of the data. Data acquisition covered two typical environments, namely orchards and packing and distribution centers, thereby providing diverse operational contexts. To sufficiently capture variations in fruit appearance, data collection was conducted from May to September, when citrus diseases are most prevalent. Images were collected for five citrus conditions, including color variation, melanosis, black spots, canker, and healthy samples (James et al., 2024; Wilfrido Gómez-Flores et al., 2024). During image acquisition, different shooting distances were adjusted to simulate near-, medium-, and long-range views, while multiple fruit orientations, including frontal, lateral, and rear views, were captured. In addition, various imaging conditions were considered, such as front lighting, backlighting, and different occlusion scenarios, thereby enhancing dataset diversity. To realistically reflect the complexity of orchard environments, the dataset includes images with natural backgrounds composed of ground surfaces, sky, and branches and leaves, as well as scenes containing both single-fruit and multi-fruit targets. As a result, a comprehensive image dataset was established that effectively reflects the diversity of natural environments and is well suited for citrus recognition and disease detection research under complex real-world conditions.

### 2.2 JXDF data preprocessing

Subsequently, data preprocessing was performed on the collected raw images. First, blurred and unclear images caused by camera shake were removed in batches. In addition, a manual inspection process was conducted to further eliminate invalid samples, including images with severe backlighting that resulted in significant feature loss, as well as samples in which targets were excessively occluded and could not be reliably identified. After systematic filtering, a total of 1, 350 high-quality citrus fruit images were retained. The LabelMe annotation tool was employed to perform detailed category labeling of the citrus images into five classes, namely healthy fruit, canker, anthracnose, scab, and black spot, and the corresponding annotation files were generated in TXT format. To ensure the consistency and reliability of the annotations, the following labeling criteria were established. (1) During annotation, bounding boxes were required to tightly enclose the target to ensure precise localization of citrus fruits. (2) Citrus categories were determined based on peel color uniformity, the morphological characteristics of disease lesions (e.g., spot-like, patch-like, or sunken areas), and the clarity of lesion boundaries. (3) For partially occluded targets, only the visible regions were annotated. (4) In cases involving multiple disease symptoms or ambiguous visual characteristics, annotations were finalized by domain experts to ensure objectivity and consistency. After comprehensive annotation, the dataset was randomly divided into training, validation, and test sets at a ratio of 8:1:1. Specifically, the training set contained 1, 080 images, while the validation and test sets each consisted of 135 images.

### 2.3 JXDF dataset characteristics analysis

In navel orange and citrus cultivation regions, fruit diseases typically exhibit a progressive transition from localized lesions to more extensive manifestations. Therefore, collecting citrus fruit data from multiple viewpoints, including frontal, lateral, and rear views, enables a more comprehensive distinction between diseased and healthy fruits, thereby ensuring reliable quality assessment for harvesting robots. The annotation criteria established in this study not only guarantee effective discrimination among the five categories, namely healthy fruit, canker, anthracnose, scab, and black spot, but also provide a comprehensive foundation for citrus tree health assessment in orchard management. Moreover, the dataset incorporates images captured under both complex natural orchard backgrounds and relatively clean backgrounds from packing and distribution centers, which helps ensure the generalization capability and environmental robustness of the model across diverse environments and background conditions. This design further enhances the scene adaptability and practical applicability of harvesting robots.

**Figure 1:**
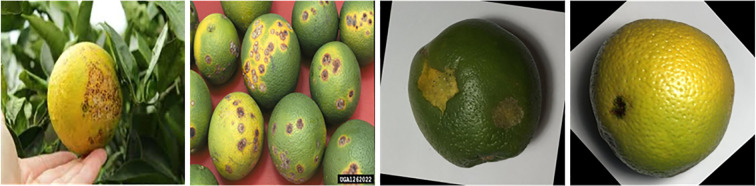
Representative examples of the JXDF dataset.

**Figure 2:**
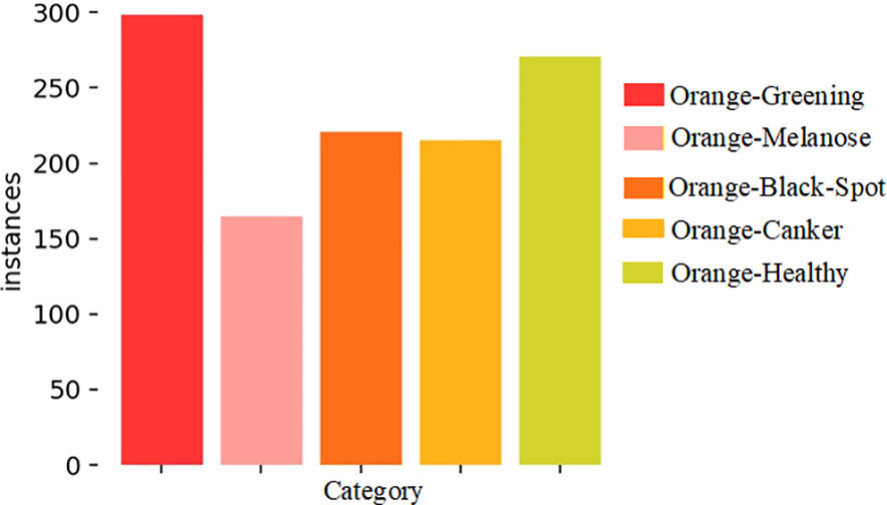
Statistical distribution of label counts for each category.

As illustrated in **Figure 1**, the constructed dataset exhibits several challenging characteristics, including complex backgrounds, frequent mutual occlusion and stacking among targets, and relatively small disease-affected regions. These characteristics indicate that the dataset can accurately reflect the typical conditions encountered in real-world navel orange and citrus disease detection scenarios and is therefore well suited to the experimental requirements of this study. The statistical distribution of category labels is shown in **Figure 2**. The dataset contains five categories Orange-Greening, Orange-Melanose, Orange-Black-Spot, Orange-Canker, and Orange-Healthy with a relatively balanced number of samples. Among them, Orange-Greening has the largest number of disease labels (300), whereas Orange-Melanose has the fewest. This distribution is consistent with the actual incidence patterns of citrus diseases observed in the field and mirrors the typical occurrence of diseases in navel orange and citrus orchards. Such a distribution helps to reduce training bias, enabling the model to learn features across all citrus categories in a balanced manner and providing a solid data foundation for constructing a robust and generalizable citrus disease recognition model.

## 3 LBM detection transformer

**Figure 3:**
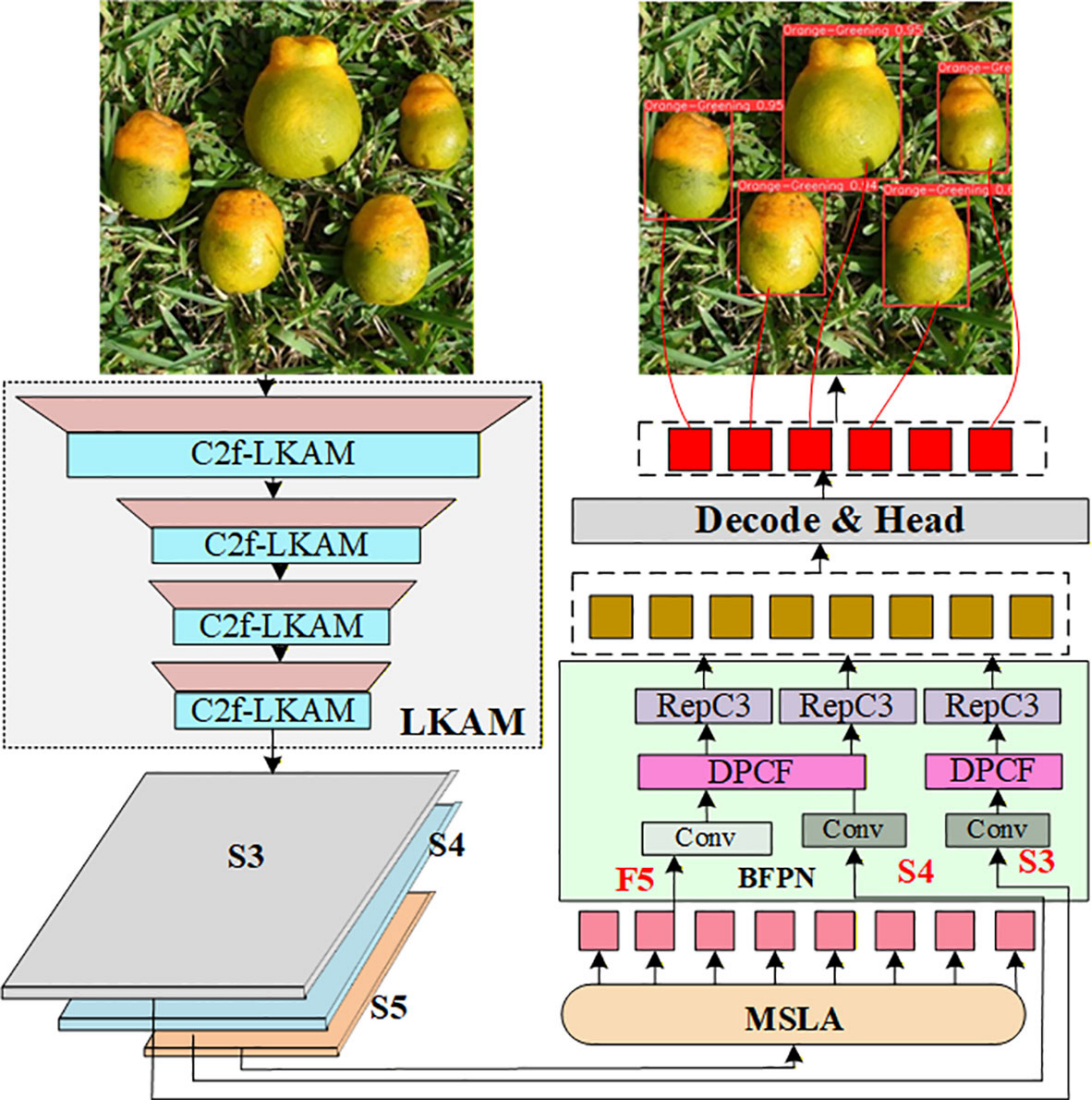
Overall network architecture diagram of the LBM detection transformer.

The RT-DETR object detection algorithm (Zhao et al., 2024) demonstrates notable advantages in real-time detection speed, accuracy, and robustness. In this study, RT-DETR is adopted as the baseline model. To achieve stable and accurate citrus detection and screening under complex cultivation environments involving occlusion, disease interference, and illumination variation, a citrus target detection algorithm based on the LBM Detection Transformer is proposed, and its overall architecture is illustrated in **Figure 3**. The proposed algorithm mainly consists of three components, namely an LKAM-based feature extraction module, an MSLA-based feature focusing module, and a BFPN-based multi-scale feature interaction and fusion module. First, citrus images collected from complex environments are fed into the LKAM module. By employing large convolutional kernels at different scales, LKAM extracts rich multi-scale features, including horizontal, vertical, texture, and edge information, and outputs comprehensive feature maps from the last three stages, denoted as S3, S4, and S5. Subsequently, the feature-rich S5 map is input into the MSLA Transformer encoder, where parallel depthwise convolutions are utilized to adaptively fuse features with different receptive fields. This design enables the network to focus more accurately on citrus targets while effectively suppressing background interference, thereby producing a refined F5 feature representation. Furthermore, the S3, S4, and F5 features are jointly fed into the BFPN module, which employs a bidirectional weighted feature fusion mechanism to efficiently integrate shallow and deep features across different levels, thereby alleviating feature attenuation caused by occlusion. Final, the fused features are passed to the detection head, producing the final citrus detection results.

Therefore, the proposed LBM Detection Transformer effectively addresses the key challenges of citrus detection in complex environments by coordinating the integration of LKAM, BFPN, and the MSLA Transformer encoder. While maintaining real-time performance, the proposed method achieves high detection accuracy and strong robustness, enabling reliable citrus target detection under challenging real-world conditions.

### 3.1 LKAM

**Figure 4:**
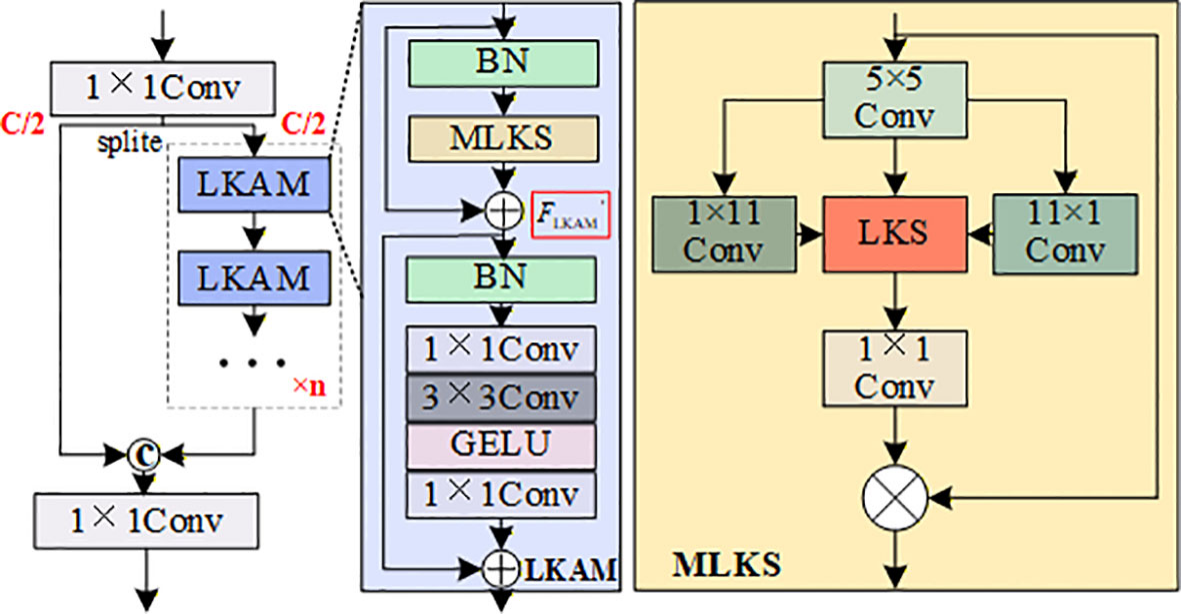
LKAM network architecture diagram.

To extract rich and comprehensive citrus features while reducing interference from complex backgrounds and occlusions, and given that a single large-kernel convolution with a fixed scale produces overly smooth features that fail to clearly separate healthy peel from diseased regions and may increase mechanical damage to healthy fruits during harvesting, we propose a large-kernel attention module (LKAM) inspired by BEVANet (Huang et al., 2025) (bilateral efficient visual attention network). The overall architecture of LKAM is illustrated in **Figure 4**. The module consists of cascaded LKAM blocks with skip connections, which are arranged in parallel with the input to enable hierarchical feature extraction from shallow to deep layers. The internal structure of LKAM is also shown in **Figure 4**. Specifically, the input feature is first processed by a batch normalization (BN) layer and a multi-scale large-kernel selection (MLKS) module, followed by a skip connection with the original input. This design achieves multi-scale weighted fusion of the original features and generates a skip-connected feature representation, denoted as $F_{\text{LKAM}}^{\prime }$. Subsequently, feature $F_{\text{LKAM}}^{\prime }$ is passed through a sequence of BN, 1×1 convolution, 3×3 convolution, GELU activation, and another 1×1 convolution to extract rich contextual and detailed features, thereby effectively suppressing background interference. Finally, the processed features are fused with the skip-connected original features to generate enhanced citrus target representations with improved richness and discriminability.

The LKAM computes the input feature *X* as follows as in Equations 1, 2:

(1) $$F_{\text{LKAM}}^{\prime }=f_{\text{BN}}\left(f_{\text{MLKS}}\left(X\right)\right)+X$$

(2) $$F_{\text{LKAM}}=f_{\text{BN}}\left(f_{1\times 1\text{Cov}}\left(\epsilon \left(f_{3\times 3\text{Cov}}\left(f_{1\times 1\text{Cov}}\left(F_{\text{LKAM}}^{\prime }\right)\right)\right)\right)\right)+F_{\text{LKAM}}^{\prime }$$

where *F*_LKAM_ denotes the output feature of LKAM, $F_{\text{LKAM}}^{\prime }$ represents the intermediate feature computed during the LKAM processing. *f*_BN_ represents batch normalization, *f*_1×1Conv_ denotes 1×1 convolution, *f*_3×2Cov_ denotes 3×3 convolution, and *ϵ* represents the GELU activation function.

**Figure 5:**
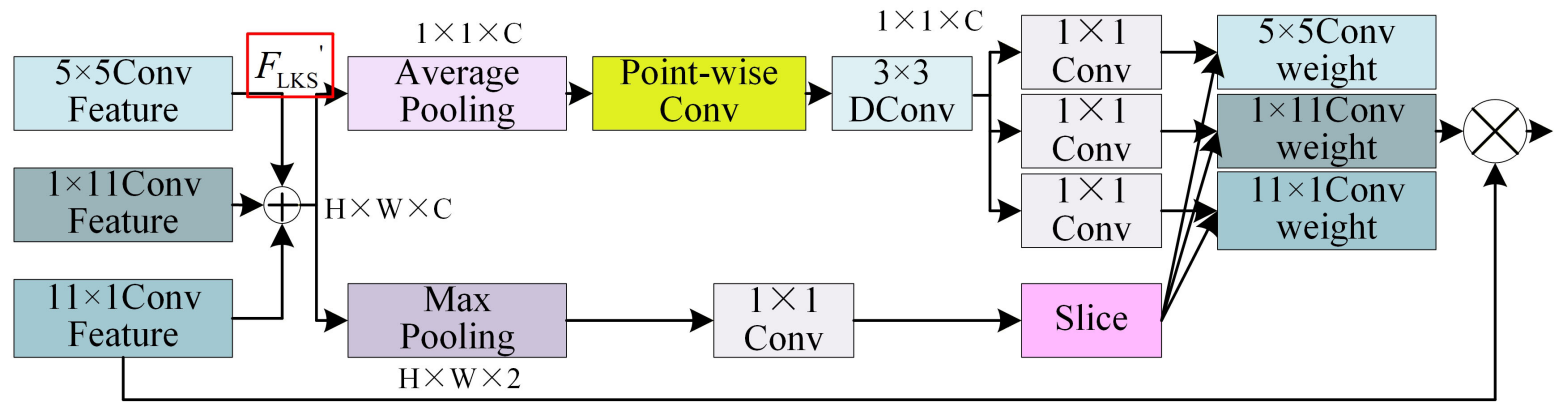
Overall architecture of the LKS network.

The network architecture of the MLSK is illustrated in **Figure 4**. This module extracts and fuses multi-scale features by employing convolutional layers with different kernel sizes. First, the input feature *X* is processed by a 5×5 convolution to obtain features with a large receptive field, enabling the network to capture rich and diverse citrus characteristics. Subsequently, the extracted features are fed into parallel branches consisting of a 1×11 convolution, a large-kernel selection (LKS) mechanism, and an 11×1 convolution, the resulting features are fused to generate a globally attentive representation. Finally, a 1×1 convolution is applied to adjust the feature dimensionality, and the output is fused with the input feature *X*, producing the final feature representation computed by the MLSK module. This network design fully considers global context, texture information, and large receptive field characteristics of citrus fruits, effectively mitigating the loss of small-target citrus features during feature extraction. The MLSK module computes the input feature *X* as follows as in Equation 3:

(3) $$F_{\text{MLKS}}=f_{\text{LKS}}[f_{1\times 11\text{Cov}}\left(f_{5\times 5\text{Cov}}\left(X\right)\right);f_{5\times 5\text{Cov}}\left(X\right);f_{11\times 1\text{Cov}}\left(f_{5\times 5\text{Cov}}\left(X\right)\right)$$

here, *f*_LKS_[;] represents the feature obtained by aggregating multiple inputs fed into the LKS module. The structure of the LKS module is illustrated in **Figure 5**. This module performs weighted feature fusion along different dimensions through three parallel branches, namely a 1×11 convolution, the LKS branch, and an 11×1 convolution. Specifically, in the first branch, the feature $F_{\text{LKS}}^{\prime }$ is successively processed by an average pooling layer, a point-wise convolution, and a 3×3 convolution. Subsequently, a 1×1 convolution is applied to adjust the feature dimensionality, producing a convolutional weight representation denoted as $F_{\text{LKS}}^{\prime }$ as in Equations 4, 5.

(4) $$F_{\text{LKS}}^{\prime }=F_{1\times 11\text{Cov}}\left(X\right)+F_{11\times 1\text{Cov}}\left(X\right)+F_{5\times 5\text{Cov}}\left(X\right)$$

(5) $$w_{1\times 11\text{Cov},11\times 1\text{Cov},5\times 5\text{Cov}}=f_{1\times 1\text{Cov}}\left(f_{3\times 3\text{DCov}}\left(f_{\text{PCov}}\left(f_{\text{AvgPool}}\left(F_{\text{LKS}}^{\prime }\right)\right)\right)\right)$$

where $w_{1\times 11\text{Cov},11\times 1\text{Cov},5\times 5\text{Cov}}$ denotes the weights corresponding to the features extracted by the 1×11, 11×1, and 5×5 convolutions, *f*_3×3DCov_ represents 3×3 depthwise separable convolution, *f*_PCov_ denotes a point-wise convolution, and *f*_AvgPool_ represents average pooling.

Subsequently, in the second branch, the feature $F_{\text{LKS}}^{\prime }$ is processed by a max-pooling layer followed by a 1×1 convolution. The resulting feature is then subjected to a slicing operation, and weighted fusion with the convolutional weight features *w* computed from the first branch is performed. Finally, in the third branch, a hadamard product is performed between the branch output and the fused features obtained from the previous stage, producing the final output feature of the LKS module as in Equation 6.

(6) $$F_{\text{LKS}}=\left(w_{5\times 5\text{Cov}}\cdot F_{5\times 5\text{Cov}}+w_{1\times 11\text{Cov}}\cdot F_{1\times 11\text{Cov}}+w_{11\times 1\text{Cov}}\cdot F_{11\times 1\text{Cov}}\right)⊙F_{1\times 11\text{Cov}}$$

Here, denotes the dot product, and ⊙ denotes the Hadamard product.

### 3.2 BFPN

**Figure 6:**
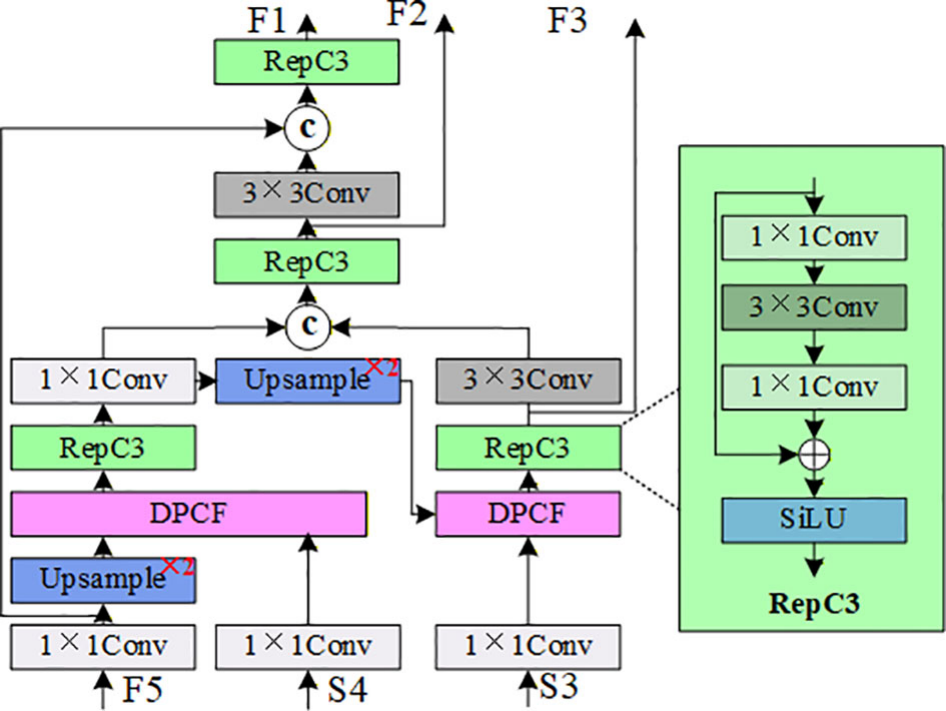
Architecture of the BFPN module.

To alleviate feature attenuation of occluded targets during network training, a BFPN is designed, inspired by SAMamba (adaptive state space mamba) (Xu et al., 2025) and the feature pyramid network (FPN) (Yang et al., 2024). The overall architecture of BFPN is illustrated in **Figure 6**. The module first takes the S3, S4, and F5 features as inputs and applies 1×1 convolutions to align channel dimensions. Subsequently, the F5 feature is upsampled to match the spatial resolution of the shallow features. The processed features are then fused in a cross-layer manner through DPCF (detail-preserving contextual fusion). Afterward, alternating RepC3 (representational C3) blocks (Vijayakumar and Vairavasundaram, 2024) and convolutional layers are employed to generate the fused feature outputs F1, F2, and F3. By adopting a bidirectional cross-connection strategy combined with an adaptive weighted fusion mechanism, BFPN efficiently integrates fine-grained details from shallow layers with high-level semantic information from deep layers. This design effectively compensates for citrus features that are lost or attenuated due to occlusion, enhances feature representation capability, and significantly improves detection accuracy and robustness in complex occlusion scenarios.

The network architecture of the RepC3 module is illustrated in **Figure 6**. RepC3 processes the input feature through a sequence of 1×1 convolution, 3×3 convolution, and 1×1 convolution. Subsequently, a skip connection is employed to fuse the transformed features with the original input. The fused feature is then passed through a SiLU activation function, producing the final output feature of the RepC3 module as in Equation 7.

(7) $$F_{\text{RepC}3}=\xi \left(X+f_{1\times 1\text{Cov}}\left(f_{3\times 3\text{Cov}}\left(f_{1\times 1\text{Cov}}\left(X\right)\right)\right)\right)$$

where *ξ* denotes the SiLU activation function.

**Figure 7:**
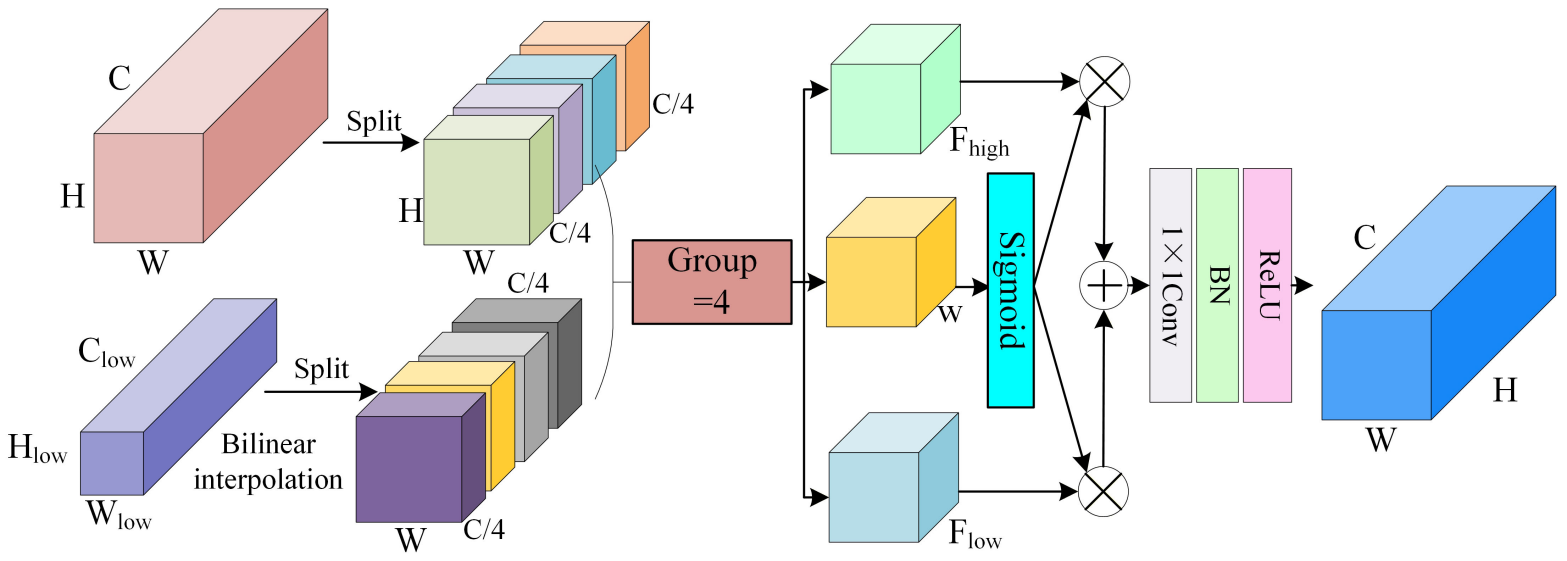
DPCF module structure diagram.

The DPCF module takes shallow and deep features with different dimensionalities as inputs. By adopting a learnable strategy to adaptively combine multi-scale features and employing a gating mechanism, DPCF achieves deep fusion of shallow and deep representations, thereby effectively preventing information dilution of deep features caused by occlusion during the fusion process. The overall workflow of the DPCF module is illustrated in **Figure 7**. Specifically, two input features from shallow and deep layers are first split into four groups along the channel dimension C. The segmented shallow and deep features are then paired to form four feature groups, in which the shallow and deep representations are respectively enhanced. Subsequently, fusion weights for shallow and deep features are computed using a Sigmoid function. Finally, the shallow and deep features are fused in a weighted manner, followed by a 1×1 convolution, batch normalization (BN), and ReLU activation. After concatenation, the module outputs the weighted fused feature representation.

The DPCF module first splits the input features *F_high_* and *F_low_* into four parts along the channel dimension C as in Equations 8, 9:

(8) $$F_{high}=\left[F_{high,1}^{\prime },F_{high,2}^{\prime },F_{high,3}^{\prime },F_{high,4}^{\prime }\right]$$

(9) $$F_{low}=\left[F_{low,1}^{\prime },F_{low,2}^{\prime },F_{low,3}^{\prime },F_{low,4}^{\prime }\right]$$

The segmented shallow and deep features are then paired in a two-by-two manner to form four feature groups as in Equation 10:

(10) $$F_{new}=\left[F_{high,1}^{\prime },F_{low,1}^{\prime }\right],\left[F_{high,2}^{\prime },F_{low,2}^{\prime }\right],\left[F_{high,3}^{\prime },F_{low,3}^{\prime }\right],\left[F_{high,4}^{\prime },F_{low,4}^{\prime }\right]$$

where *F_high_* denotes the deep feature, *F_low_* denotes the shallow feature, $\left[F_{high,1}^{\prime },F_{high,2}^{\prime },F_{high,3}^{\prime },F_{high,4}^{\prime }\right]$ represents the four feature segments obtained by splitting along the channel dimension C, and *F_new_* denotes the regrouped features. Taking the combined feature $\left[F_{high,1}^{\prime },F_{low,1}^{\prime }\right]$ as an example, learnable feature weights are computed. The weighted fused feature *F_fusion_* is calculated as follows as in Equations 11, 12:

(11) $$F_{fusion}=w_{high}\cdot F_{high}+\left(1-w_{high}\right)\cdot F_{low}$$

(12) $$w_{high}=\alpha \cdot F_{high},\;F_{high}\in {ℛ}^{H\times W\times C/4}$$

where *F_fusion_* denotes the fused feature, *w_high_* represents the learnable weights of the channel-wise feature *F_high_*, and *α* denotes the Sigmoid activation function. The DPCF module outputs the feature as follows as in Equation 13:

(13) $$F_{\text{DPCF}}=\delta \left(f_{\text{BN}}\left(f_{1\times 1\text{Cov}}\left(F_{fusion}\right)\right)\right)$$

where *δ* denotes the ReLU activation function.

The design of BFPN follows the principle of efficient multi-scale feature fusion. By constructing a bidirectional multi-scale fusion pathway together with an adaptive weighting mechanism, BFPN enables dynamically selectable feature fusion. During citrus detection, the module adaptively selects and fuses input features, thereby effectively enhancing feature integration under complex backgrounds, occluded targets, and small-scale citrus instances. As a result, BFPN improves the model’s detection performance for citrus targets with subtle disease characteristics in complex scenarios.

### 3.3 MSLA transformer encoder

**Figure 8:**
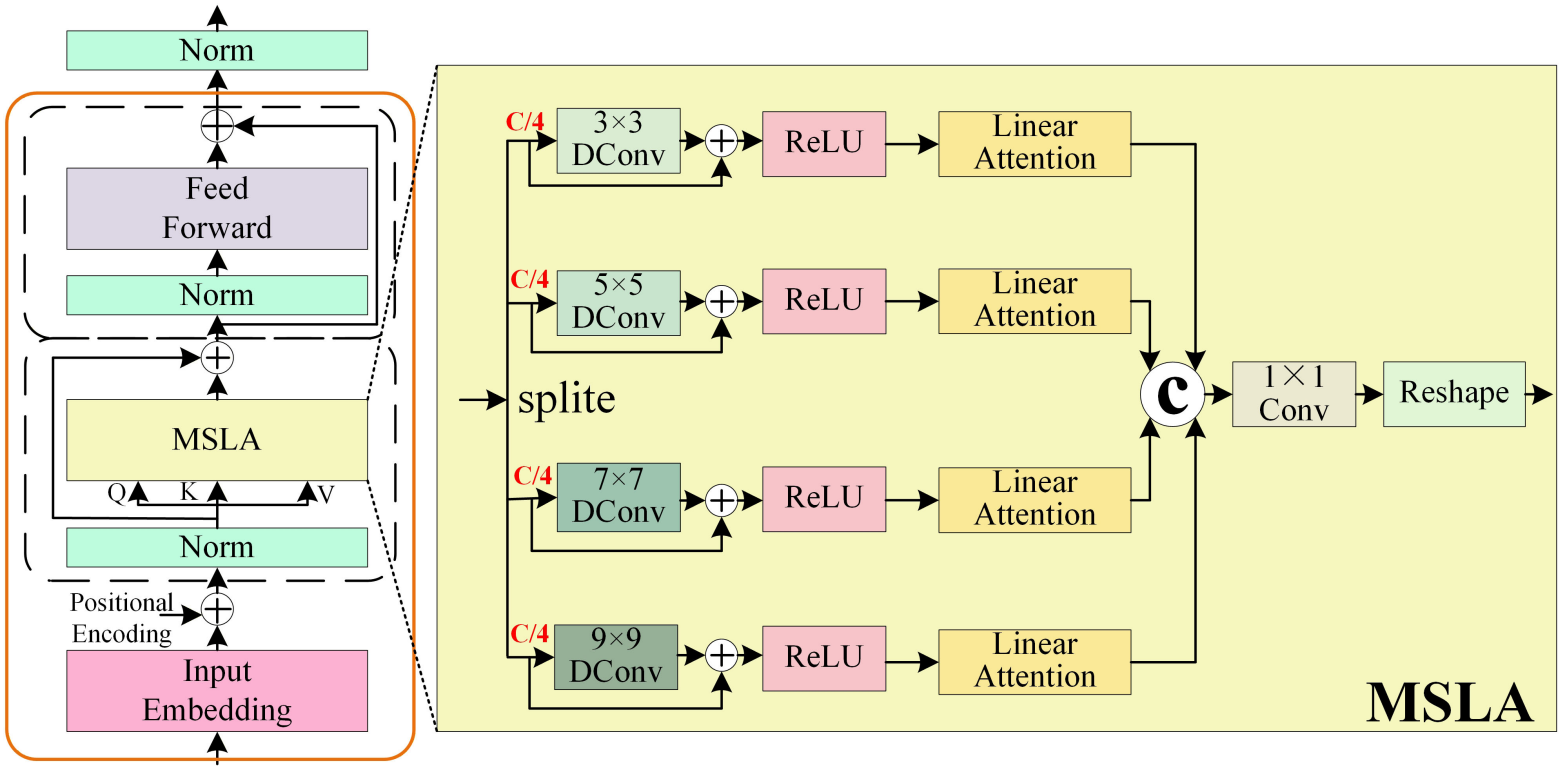
Structure of MSLA transformer encoder module.

Traditional Transformer attention methods perform interactive computations over all regions, including complex citrus backgrounds, which prevents selective focus on diseased areas and increases both false positives and false negatives in citrus disease detection. An MSLA Transformer encoder is designed, inspired by MSLAU-Net (a novel hybrid CNN–transformer architecture) (Lan et al., 2025) and the standard Transformer framework (Vaswani et al., 2017). By enhancing the deep feature representation of F5 across multiple scales, the proposed encoder enables the network to focus more accurately on citrus target features, thereby reducing undesired interactions with complex background information. The network architecture of the MSLA Transformer encoder is illustrated in **Figure 8**.

The MSLA Transformer encoder follows the standard design paradigm of a Transformer encoder. First, the input features are normalized and projected through a linear mapping layer. Subsequently, the key *K*, query *Q*, and value *V* matrices are computed, and multi-scale linear attention (MSLK) is applied to enhance feature representations, enabling effective aggregation of global citrus target information. Finally, a feed-forward network followed by normalization and linear layer is applied, together with a residual connection to the input features, thereby producing more focused and enhanced feature representations. The structure of MSLA is illustrated in **Figure 8**. In the standard Transformer encoder, self-attention is employed to model global dependencies and enhance deep feature representations. However, self-attention requires the separate computation of *K*, *Q*, and *V* matrices, followed by complex matrix multiplications to model their interactions, which substantially increases computational overhead. Moreover, self-attention performs dense global encoding over all spatial positions, inevitably introducing complex background information during the feature focusing process and thereby reducing the discriminative capability of the network. In contrast, MSLA leverages multi-scale feature representations and linear additive operations to achieve feature fusion. This design not only reduces computational complexity but also enables the network to focus more effectively on citrus target features while decreasing sensitivity to complex backgrounds, thereby resulting in more efficient feature interaction.

Specifically, MSLA splits the input feature *X* along the channel dimension and performs parallel computations to accelerate network training as in Equations 14–18:

(14) $$F_{\text{splite}}=\left[X_{1}^{\prime },X_{2}^{\prime },X_{3}^{\prime },X_{4}^{\prime }\right]$$

(15) $$F_{3\times 3\text{DCov}}=\delta \left(f_{3\times 3\text{DCov}}\left(X_{1}^{\prime }\right)+X_{1}^{\prime }\right)$$

(16) $$F_{5\times 5\text{DCov}}=\delta \left(f_{5\times 5\text{DCov}}\left(X_{2}^{\prime }\right)+X_{2}^{\prime }\right)$$

(17) $$F_{7\times 7\text{DCov}}=\delta \left(f_{7\times 7\text{DCov}}\left(X_{3}^{\prime }\right)+X_{3}^{\prime }\right)$$

(18) $$F_{9\times 9\text{DCov}}=\delta \left(f_{9\times 9\text{DCov}}\left(X_{4}^{\prime }\right)+X_{4}^{\prime }\right)$$

MSLA concatenates the multi scale features and applies a 1×1 convolution to adjust the feature dimensionality, as formulated below as in Equation 19:

(19) $$F_{\text{MSLA}}=f_{1\times 1\text{Cov}}\left[F_{3\times 3\text{DCov}};F_{5\times 5\text{DCov}};F_{7\times 7\text{DCov}};F_{9\times 9\text{DCov}}\right]$$

where [;] denotes the concatenation operation.

## 4 Experimental results and analysis

### 4.1 Experimental environment

All experiments in this study were conducted on a Windows 11 operating system. The hardware platform was equipped with an NVIDIA GeForce RTX 4080 Super GPU, an Intel Core i7-13700K processor operating at 3.40 GHz, and 32 GB of RAM. Model construction, training, and evaluation were implemented in Python 3.10 within the PyCharm development environment. The deep learning framework used was PyTorch 2.2.2, and the primary libraries included Ultralytics, OpenCV, NumPy, and Pillow.

For model training, the input image resolution was set to 640×640 pixels, the batch size was set to 8, and the total number of training epochs was set to 200. The Adam optimizer was adopted for stochastic gradient descent, with a momentum parameter of 0.937, an initial learning rate of 0.001, and a weight decay coefficient of 0.0005. To evaluate inference efficiency, frames per second (FPS) was measured using a batch size of 1. In addition, all models were trained with randomly initialized weights without using any pretrained parameters. This approach was adopted to eliminate biases in feature extraction arising from external dataset priors, ensuring that performance differences on the current dataset were solely attributable to the network architecture and optimization strategies rather than domain adaptation effects from pretrained weights. Such a design guarantees fair comparison among models and reproducibility of the experimental results.

### 4.2 Evaluation metrics

For detection accuracy evaluation, average precision (AP) for each category, mean average precision (mAP) across all categories, mmAP under different intersection over union (IoU) thresholds (IoU = 0.5:0.05:0.95), and recall (R) are adopted as evaluation metrics. Specifically, AP, mAP, and mmAP are used to assess the false detection behavior of the algorithm, while R reflects the missed detection rate. For all four metrics, higher values indicate better detection accuracy and stability, corresponding to fewer false positives and false negatives. In addition to detection accuracy, model size and real-time inference performance are also critical for citrus target detection. Therefore, the number of parameters (Para), GFLOPs, and detection speed measured in frames per second (FPS) are employed for efficiency evaluation. The number of parameters and GFLOPs characterize the structural complexity and computational scale of the model. A smaller model typically contains fewer parameters and lighter weights, enabling faster training and inference as well as more efficient lightweight deployment. FPS is commonly used to evaluate the computational efficiency of object detection algorithms and reflects the detection speed on a given hardware platform. Real-time detection is generally achieved when FPS≥30, and higher FPS values indicate better real-time performance (Yun et al., 2022).

### 4.3 Ablation study

To verify the effectiveness of the proposed improvement strategies in the LBM Detection Transformer, ablation experiments were conducted on the JXDF dataset under identical experimental conditions. RT-DETR was adopted as the baseline model. The variant incorporating the LKAM module is denoted as M1, the variant integrating the MSLA Transformer encoder is denoted as M2, and the variant employing the BFPN module is denoted as M3. All ablation experiments were performed using the same dataset and experimental settings, and the corresponding results are summarized in **Table 1**.

**Table 1:** Ablation comparison of AP for each category.

Class	AP/%			
	RT-DETR	RT-DETR+M1	RT-DETR+M1+M2	RT-DETR+M1+M2+M3
Orange-Greening	92.49	94.84	95.96	**98.14**
Orange-Melanose	94.01	95.15	95.49	**97.26**
Orange-Black-Spot	94.17	96.84	95.63	**97.11**
Orange-Canker	93.78	96.53	97.71	**99.53**
Orange-Healthy	91.39	93.44	94.89	**97.25**
mAP	93.17	95.36	95.94	**97.86**

Bold indicates the optimal value.

The per-category AP ablation results presented in **Table 1** demonstrate that all three proposed optimization strategies lead to varying degrees of performance improvement across the five target categories. The proposed LBM Detection Transformer achieves the highest AP values for all categories, reaching 98.14%, 97.26%, 97.11%, 99.53%, and 97.25% for Orange-Greening, Orange-Melanose, Orange-Blackspot, Orange-Canker, and Orange-Healthy, respectively. Compared with the baseline RT-DETR, the proposed method improves mAP by 4.69%. These results indicate that the improvements introduced by LKAM, the MSLA Transformer encoder, and BFPN jointly contribute to enhanced detection accuracy. By effectively addressing challenges such as occlusion, disease interference, and illumination variation in complex citrus cultivation environments, the proposed method enables stable and accurate citrus detection and screening.

The ablation results for the remaining evaluation metrics are reported in **Table 2**, showing that all three optimization strategies contribute positively to overall performance. The proposed LBM Detection Transformer achieves the best performance across mAP, mmAP, and R, reaching 97.86%, 93.88%, and 95.14%, respectively. This demonstrates that the LKAM module, through multi-scale large-kernel convolutions, simultaneously captures global contours, local edges, and texture information, effectively suppressing feature interference caused by complex backgrounds and partial occlusions. With LKAM, mAP, mmAP, and R are improved by 2.19%, 2.09%, and 1.57%, respectively. Meanwhile, LKAM employs depthwise separable large-kernel convolutions for a lightweight design, reducing parameters by 5.38M and GFLOPs by 8.25. The MSLA Transformer encoder adopts a linear attention mechanism, replacing the matrix dot product in traditional multi-head self-attention with additive operations, which reduces the encoder parameters and GFLOPs by 0.17M and 0.05, respectively. Additionally, by performing multi-scale feature interaction, the MSLA encoder establishes dense connections across convolution outputs with different receptive fields, effectively mitigating feature attenuation for occluded targets in deeper layers. The BFPN module employs a block-wise parallel bidirectional fusion strategy, introducing adaptively weighted interactions along both top-down and bottom-up paths, deeply integrating shallow detailed features with deep semantic information. This results in increases of 1.92%, 2.61%, and 1.25% in mAP, mmAP, and R, respectively.

**Table 2:** Ablation comparison of different improvement strategies.

RT-DETR	M1	M2	M3	mAP/%	mmAP/%	R/%	Para/M	GFLOPs	FPS frame/s
✓				93.17	88.74	91.48	20.09	58.34	85.71
✓	✓			95.36	90.83	93.05	14.71	50.09	92.25
✓	✓	✓		95.94	91.27	93.89	**14.54**	**50.04**	**93.28**
✓	✓	✓	✓	**97.86**	**93.88**	**95.14**	14.55	50.05	92.77

Bold indicates the optimal value.

In summary, the LBM Detection Transformer adopts a lightweight network design overall. By integrating the three proposed modules, the model achieves the highest accuracy and efficiency on the test set, with parameters reduced from 20.09M to 14.55M (a reduction of approximately 28%) and GFLOPs decreased from 58.34 to 50.05. At the same time, mAP improves by 4.69%, mmAP by 5.14%, and R by 3.66%, demonstrating that the network achieves both high detection accuracy and lightweight design.

**Figure 9:**
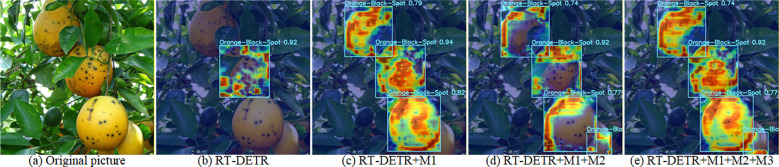
Comparison of thermal diagram of ablation experiment.

To intuitively illustrate differences in attention to citrus targets under different optimization strategies, a comparison of heatmap visualizations is presented in **Figure 9**. The comparison between **Figures 9b, c** shows that the incorporation of LKAM enables the network to attend to more comprehensive and informative citrus target features, thereby improving citrus recognition accuracy in complex scenes. The heatmap comparison between **Figures 9c, d** further indicates that the MSLA Transformer encoder effectively focuses on small and occluded citrus targets, which contributes to a reduction in missed detections. In addition, the visualization results in **Figures 9d, e** demonstrate that the introduction of the BFPN structure allows the network to focus more precisely on target regions by integrating both shallow and deep features, thereby leading to an overall improvement in detection performance. In summary, the combined evidence from quantitative evaluation metrics and heatmap-based qualitative visualizations confirms the rationality and effectiveness of the proposed optimization strategies, as well as the strong performance of the proposed method in complex citrus detection scenarios.

### 4.4 Comparative experiments

**Figure 10:**
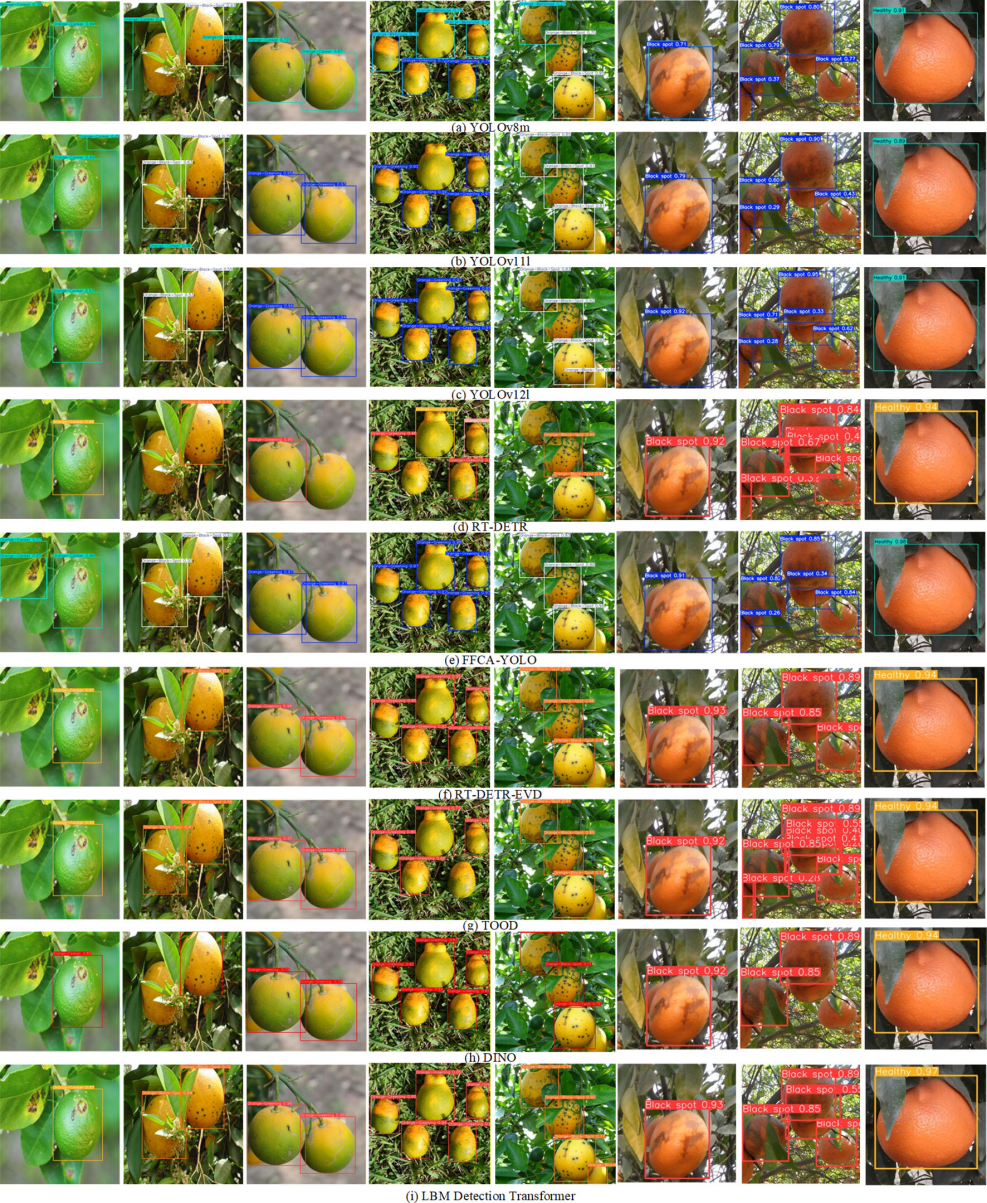
Visualization of detection results in the comparative experiments.

To verify the effectiveness and generalization capability of the proposed LBM Detection Transformer, comparative experiments were conducted on the OFDD and JXDF datasets under identical experimental conditions. Six representative state-of-the-art object detection methods with comparable model scales and performance were selected for comparison, including YOLOv8m (Sohan et al., 2024.), YOLOv11l (Jegham et al., 2025), YOLOv12l (Tian et al., 2025), RT-DETR (Zhao et al., 2024), TOOD (Feng et al., 2021), and DINO (Zhang et al., 2022), as well as two lightweight networks FFCA-YOLO (Ge et al., 2024) and RT-DETR-EVD (Hu et al., 2025). The comparative experimental results are presented in **Table 3** and **Figure 10**.

**Table 3:** Experimental comparison of different methods.

Datasets	Methods	mAP/%	mmAP/%	R/%	Para/M	GFLOPs	FPS frame/s
JXDF	YOLOv8m	94.15	91.57	89.61	25.91	78.91	88.48
	YOLOv11l	94.37	91.89	90.37	25.37	86.88	79.46
	YOLOv12l	95.88	92.41	93.19	26.44	88.92	75.59
	RT-DETR	93.17	88.74	91.48	20.09	58.34	85.71
	FFCA-YOLO	95.56	91.44	92.81	**7.12**	**27.43**	**145.85**
	RT-DETR-EVD	96.14	92.81	94.38	14.23	49.51	108.17
	TOOD	94.24	92.16	93.49	32.03	123.04	54.38
	DINO	96.32	92.59	94.86	22.24	91.70	73.24
	LBM Detection Transformer	**97.86**	**93.88**	**95.14**	14.55	50.05	92.77
OFDD	YOLOv8m	92.19	75.95	89.74	25.91	78.91	88.48
	YOLOv11l	92.76	77.18	90.42	25.37	86.88	79.46
	YOLOv12l	93.21	77.43	90.84	26.44	88.92	75.59
	RT-DETR	92.87	77.26	90.58	20.09	58.34	85.71
	FFCA-YOLO	94.04	79.33	91.51	**7.12**	**27.43**	**145.85**
	RT-DETR-EVD	94.85	80.39	92.04	14.23	49.51	108.17
	TOOD	92.14	76.48	90.07	32.03	123.04	54.38
	DINO	93.88	78.15	91.27	22.24	91.70	73.24
	LBM Detection Transformer	**95.92**	**80.77**	**93.23**	14.55	50.05	92.77

Bold indicates the optimal value.

As shown in **Table 3**, the proposed LBM Detection Transformer demonstrates superior overall performance in detection accuracy, model size, and real-time inference, achieving efficient detection of citrus targets. Since the same comparative algorithms and hyperparameters were applied across both datasets, the parameters (Para), GFLOPs, and FPS remain unchanged. On the JXDF dataset, the proposed method achieves mAP, mmAP, and R of 97.86%, 93.88%, and 95.14%, respectively, with Para, GFLOPs, and FPS ranking third overall at 14.55M, 50.05, and 92.77. Compared with the baseline RT-DETR, the proposed method reduces Para and GFLOPs by 5.54M and 8.29, while improving mAP by 4.69%, mmAP by 5.14%, and R by 3.66%, thus effectively balancing detection accuracy and computational efficiency.

On the OFDD dataset, the method achieves mAP, mmAP, and R of 95.92%, 80.77%, and 93.23%, respectively, corresponding to improvements of 3.05%, 3.51%, and 2.65% over the baseline RT-DETR. These results confirm the method’s generalization capability and applicability in citrus fruit disease detection. Comparative methods such as TOOD, DINO, and YOLOv12l also exhibit certain advantages in citrus detection accuracy. TOOD utilizes an aligned task predictor to achieve high-precision detection of small targets in complex environments; however, its single-point prediction approach increases both model parameters and computational complexity. DINO employs Transformer-based global attention combined with an end-to-end NMS-free mechanism, prioritizing precision through a global optimal strategy, but this comes at the expense of computational efficiency. YOLOv12l, when detecting small citrus targets, shows weaker adaptability in dynamic scenarios due to its label assignment strategy and loss function design, resulting in limited model generalization. Two lightweight networks, FFCA-YOLO and RT-DETR-EVD, achieve moderate accuracy and real-time efficiency. FFCA-YOLO leverages lightweight cross-scale attention and affine feature fusion, while RT-DETR-EVD employs a variable-depth encoder with query dimension reduction. These designs maintain high precision and improve inference speed; however, the lightweight architecture may excessively compress channel dimensions or prematurely prune background queries during feature focusing, causing loss of discriminative information for weak targets or blurred textures in complex backgrounds, which can lead to false negatives and false positives.

**Figure 10** presents the qualitative detection results of different methods on the citrus dataset. Specifically, the first five columns show visual detection results on the JXDF dataset: the first column depicts scenes with backgrounds similar to citrus targets, the second column contains occluded targets, the third column shows small diseased citrus targets, the fourth column presents dense and complex backgrounds, and the fifth column illustrates scenarios with mutually occluded targets. Columns six to eight display visual detection results on the OFDD dataset: the sixth column shows complex background scenes, the seventh column contains dense and mutually occluded targets, and the eighth column depicts single citrus fruits. On the JXDF dataset, in the first column (backgrounds similar to citrus targets), YOLOv8m, YOLOv11l, and FFCA-YOLO produced false positives due to background confusion, whereas YOLOv12l, RT-DETR, RT-DETR-EVD, TOOD, DINO, and the proposed LBM Detection Transformer correctly detected diseased citrus targets, with the LBM Detection Transformer achieving the highest accuracy of 97%. In the second column (occluded targets), YOLOv8m and YOLOv11l produced false positives, while RT-DETR, RT-DETR-EVD, and DINO exhibited noticeable false negatives; YOLOv12l, FFCA-YOLO, TOOD, and LBM Detection Transformer accurately detected all targets, with LBM Detection Transformer performing best. In the third column (small diseased targets), YOLOv8m produced false positives and RT-DETR missed some targets, whereas YOLOv11l, YOLOv12l, FFCA-YOLO, RT-DETR-EVD, TOOD, DINO, and LBM Detection Transformer correctly detected all targets, with LBM Detection Transformer achieving the highest accuracy. In the fourth column (dense, complex backgrounds), YOLOv8m and RT-DETR generated incorrect detection categories, YOLOv11l and TOOD exhibited false negatives, while YOLOv12l, DINO, and LBM Detection Transformer successfully detected all targets, with LBM Detection Transformer providing the most accurate detection and localization. In the fifth column (mutually occluded targets), YOLOv8m produced false positives, YOLOv11l, RT-DETR, FFCA-YOLO, RT-DETR-EVD, TOOD, and DINO exhibited varying degrees of false negatives, whereas YOLOv12l and LBM Detection Transformer correctly detected all occluded targets, with the proposed method demonstrating superior precision and localization. On the OFDD dataset, in the sixth column (complex background), YOLOv8m produced redundant overlapping bounding boxes, while the other comparative methods correctly detected diseased fruits, with LBM Detection Transformer achieving the highest precision. In the seventh column (dense and mutually occluded targets), YOLOv8m, YOLOv11l, RT-DETR-EVD, and DINO exhibited varying degrees of false negatives, RT-DETR and TOOD produced redundant false positives, whereas YOLOv12l, FFCA-YOLO, and LBM Detection Transformer correctly detected all diseased fruits without false positives or negatives. In the eighth column (single citrus fruits), all comparative methods successfully detected targets in the simple scene, with LBM Detection Transformer showing superior accuracy and novelty. Overall, the proposed LBM Detection Transformer produced no false positives or false negatives across all eight scenarios, achieving high detection accuracy and demonstrating its capability for real-time and precise detection of citrus targets.

In summary, based on comprehensive quantitative evaluations and comparative visualizations, the proposed LBM Detection Transformer enables effective, real-time, and accurate citrus target detection. The proposed method effectively alleviates false positives and missed detections caused by complex backgrounds and small disease regions, thereby improving detection performance, localization accuracy, and generalization capability.

## 5 Conclusion

To address common challenges in citrus orchards, such as fruit occlusion, small disease regions, and high similarity between background and target features, this study proposes a lightweight LBM Detection Transformer for citrus recognition. First, a multi-scale feature extraction method was designed within the LKAM, employing multi-scale large-kernel convolutions to fully capture rich citrus target features. Next, the MSLA Transformer encoder was introduced to enhance model focus on citrus regions through feature propagation and interaction. Finally, the BFPN was utilized to effectively fuse deep and shallow features, compensating for occluded citrus targets and reducing false negatives. The combination of these three modules provides complementary effects in mitigating local occlusion, reusing multi-scale semantic information, and improving spatial resolution for small targets, making it indispensable for practical applications in complex citrus orchard environments. Ablation studies verified the effectiveness and comparability of the proposed improvements in the LBM Detection Transformer. Compared with the baseline model, the proposed method reduced parameters by 5.54M, while improving mAP by 4.69%, mmAP by 5.14%, and R by 3.66%. Comparative experiments further demonstrated that the method can overcome the challenges of detecting occluded citrus targets in complex scenarios, exhibiting strong generalization capability and stable detection performance. However, experiments indicated that the LBM Detection Transformer still requires further enhancement in detecting and preventing early-stage citrus diseases. Future work will focus on detecting even smaller disease regions to prevent further spread, thereby ensuring citrus yield and quality.

## Data Availability

The datasets presented in this study can be found in online repositories. The names of the repository/repositories and accession number(s) can be found in the article/supplementary material.

## References

[B1] Chen J. Wang S. Guo J. Chen F. Li Y. Qiu H. (2024a). Improved FasterViT model for citrus disease diagnosis. Heliyon 10, e36092. doi: 10.1016/j.heliyon.2024.e36092 39247290 PMC11378920

[B2] Chen Z. Lei X. Yuan Q. Qi Y. Ma Z. Qian S. . (2024b). Key technologies for autonomous fruit- and vegetable-picking robots: A review. Agronomy-Basel 14, 2233. doi: 10.3390/agronomy14102233 30654563

[B3] Elaraby A. Hamdy W. Alanazi S. (2022). Classification of citrus diseases using optimization deep learning approach-Web of Science Core Collection. Available online at: https://webofscience.clarivate.cn/wos/woscc/full-record/WOS:000767538800009 (Accessed March 28, 2026). 10.1155/2022/9153207 PMC8853760 35186072

[B4] Faisal S. Javed K. Ali S. Alasiry A. Marzougui M. Khan M. A. . (2023). Deep transfer learning based detection and classification of citrus plant diseases. CMC-Comput. Mat. Contin. 76, 895–914. doi: 10.32604/cmc.2023.039781

[B5] Feng C. Zhong Y. Gao Y. Scott M. R. Huang W. (2021). “ TOOD: Task-aligned one-stage object detection”, in: From the 2021 IEEE/CVF International Conference on Computer Vision (ICCV 2021) (New York: IEEE), 3490–3499. doi: 10.1109/ICCV48922.2021.00349

[B6] Ge Y. Ji W. Yin S. Zhang W. (2024). “ Small object detection in remote sensing based on feature fusion and channel attention”, in: From the 2024 8th International Symposium on Computer Science and Intelligent Control, Iscsic (Los Alamitos: IEEE Computer Soc), 195–200. doi: 10.1109/ISCSIC64297.2024.00048

[B7] Gómez-Flores W. Garza-Saldaña J. J. Varela-Fuentes S. E. (2024). CitrusUAT: A dataset of orange Citrus sinensis leaves for abnormality detection using image analysis techniques. Data Brief 52, 109908. doi: 10.1016/j.dib.2023.109908 38093853 PMC10716766

[B8] Gu Y. Syafrudin M. (2025). Citrus diseases detection using innovative deep learning approach and hybrid meta-heuristic-Web of Science Core Collection. Available online at: https://webofscience.clarivate.cn/wos/woscc/full-record/WOS:001492214200026 (Accessed March 28, 2026). 10.1371/journal.pone.0316081 PMC11753642 39841644

[B9] Hou C. Xu J. Tang Y. Jiajun Z. Tan Z. Chen W. . (2024). Detection and localization of citrus picking points based on binocular vision. Precis. Agric. 25, 2321–2355. doi: 10.1007/s11119-024-10169-2 30311153

[B10] Hu X. Liao M. Duan J. Luo J. Gong P. (2024). Contour analysis and gridding-based navel orange recognition algorithm for automatic picking robot | Springer Nature Link. Available online at: https://link.springer.com/chapter/10.1007/978-981-96-3161-2_17 (Accessed March 28, 2026).

[B11] Hu J. Zheng J. Wan W. Zhou Y. Huang Z. (2025). RT-DETR-EVD: An emergency vehicle detection method based on improved RT-DETR. Sensors 25, 3327. doi: 10.3390/s25113327 40968878 PMC12157159

[B12] Huang P.-M. Chao I.-T. Huang P.-C. Liao J.-W. Chuang Y.-Y. (2025). “ Bevanet: Bilateral efficient visual attention network for real-time semantic segmentation”, in: 2025 IEEE International Conference on Image Processing (ICIP), 2778–2783. doi: 10.1109/ICIP55913.2025.11084676

[B13] James J. A. Manching H. K. Mattia M. R. Bowman K. D. Hulse-Kemp A. M. Beksi W. J. (2024). CitDet: A benchmark dataset for citrus fruit detection. IEEE Robot. Autom. Lett. 9, 10788–10795. doi: 10.1109/LRA.2024.3474473 25079929

[B14] Jegham N. Koh C. Y. Abdelatti M. Hendawi A. (2025). YOLO evolution: A comprehensive benchmark and architectural review of YOLOv12, YOLO11, and their previous versions. arXiv preprint arXiv:2411.00201. doi: 10.48550/arXiv.2411.00201

[B15] Lan L. Li Y. Liu X. Zhou J. Zhang J. Huang N. . (2025). MSLAU-Net: A hybird CNN-transformer network for medical image segmentation. doi: 10.48550/arXiv.2505.18823

[B16] Lu J. Chen W. Lan Y. Qiu X. Huang J. Luo H. (2024). Design of citrus peel defect and fruit morphology detection method based on machine vision. Comput. Electron. Agric. 219, 108721. doi: 10.1016/j.compag.2024.108721 38826717

[B17] R M S. Gladston A. Nehemiah K. H. (2025). A multi-kernel CNN model with attention mechanism for classification of citrus plants diseases. Sci. Rep. 15, 24047. doi: 10.1038/s41598-025-08557-3 40617859 PMC12228722

[B18] Sohan M. Thotakura Sai Ram Ch. Venkata Rami Reddy (2024). A review on YOLOv8 and its advancements | Springer Nature Link. Available online at: https://link.springer.com/chapter/10.1007/978-981-99-7962-2_39 (Accessed March 28, 2026).

[B19] Tang Y. Chen M. Wang C. Luo L. Li J. Lian G. . (2020). Recognition and localization methods for vision-based fruit picking robots: A review. Front. Plant Sci. 11, 510. doi: 10.3389/fpls.2020.00510 32508853 PMC7250149

[B20] Tian Y. Ye Q. Doermann D. (2025). YOLOv12: Attention-centric real-time object detectors. doi: 10.48550/arXiv.2502.12524

[B21] Vaswani A. Shazeer N. Parmar N. Uszkoreit J. Jones L. Gomez A. N. . (2017). “ Attention is all you need”, in: From the Advances in Neural Information Processing Systems 30 (nips 2017) (La Jolla: Neural Information Processing Systems (NIPS).

[B22] Vijayakumar A. Vairavasundaram S. (2024). YOLO-based object detection models: A review and its applications. Multimed. Tools Appl. 83, 83535–83574. doi: 10.1007/s11042-024-18872-y 30311153

[B23] Wang C. Pan W. Zou T. Li C. Han Q. Wang H. . (2024). A review of perception technologies for berry fruit-picking robots: Advantages, disadvantages, challenges, and prospects. Agriculture-Basel 14, 1346. doi: 10.3390/agriculture14081346 30654563

[B24] Xiao X. Jiang Y. Wang Y. (2024a). A method of robot picking citrus based on 3D detection. IEEE Instrum. Meas. Mag. 27, 50–58. doi: 10.1109/MIM.2024.10505191 25079929

[B25] Xiao X. Wang Y. Jiang Y. Wu H. Zhou B. (2024b). Monocular pose estimation method for automatic citrus harvesting using semantic segmentation and rotating target detection. Foods 13, 2208. doi: 10.3390/foods13142208 39063292 PMC11276354

[B26] Xiao X. Wang Y. Zhou B. Jiang Y. (2024c). Flexible hand claw picking method for citrus-picking robot based on target fruit recognition. Agriculture-Basel 14, 1227. doi: 10.3390/agriculture14081227 30654563

[B27] Xie W. Feng F. Zhang H. (2024). A detection algorithm for citrus huanglongbing disease based on an improved YOLOv8n-Web of Science Core Collection. Available online at: https://webofscience.clarivate.cn/wos/woscc/full-record/WOS:001277601700001 (Accessed March 28, 2026). 10.3390/s24144448 PMC11280536 39065846

[B28] Xu W. Zheng S. Wang C. Zhang Z. Ren C. Xu R. . (2025). SAMamba: Adaptive state space modeling with hierarchical vision for infrared small target detection. Inf. Fusion 124, 103338. doi: 10.1016/j.inffus.2025.103338 38826717

[B29] Xue R. Wang L. (2025). Research on lightweight citrus leaf pest and disease detection based on PEW-YOLO. Processes 13, 1365. doi: 10.3390/pr13051365 30654563

[B30] Yang G. Lei J. Tian H. Feng Z. Liang R. (2024). Asymptotic feature pyramid network for labeling pixels and regions. IEEE Trans. Circuits Syst. Video Technol. 34, 7820–7829. doi: 10.1109/TCSVT.2024.3376773 25079929

[B31] Ye L. Ma J. Lv Y. Guo Z. Lai Z. Ou C. . (2025). The YOLO-OBB-based approach for citrus fruit stem pose estimation and robot picking. Agriculture-Basel 15, 2330. doi: 10.3390/agriculture15222330 30654563

[B32] Yun J. Jiang D. Liu Y. Sun Y. Tao B. Kong J. . (2022). Real-time target detection method based on lightweight convolutional neural network. Front. Bioeng. Biotechnol. 10, 861286. doi: 10.3389/fbioe.2022.861286 36051585 PMC9426345

[B33] Zhang H. Ji W. Xu B. Yu X. (2024b). Optimizing contact force on an apple picking robot end-effector. Agriculture-Basel 14, 996. doi: 10.3390/agriculture14070996 30654563

[B34] Zhang H. Li F. Liu S. Zhang L. Su H. Zhu J. . (2022). DINO: DETR with improved denoising anchor boxes for end-to-end object detection. doi: 10.48550/arXiv.2203.03605

[B35] Zhang B. Yin C. Fu Y. Xia Y. Fu W. (2024a). Harvest motion planning for mango picking robot based on improved RRT-Connect. Biosyst. Eng. 248, 177–189. doi: 10.1016/j.biosystemseng.2024.10.008 38826717

[B36] Zhao Y. Lv W. Xu S. Wei J. Wang G. Dan Q. . (2024). “ DETRs beat YOLOs on real-time object detection”, in: From the 2024 Ieee/Cvf Conference on Computer Vision and Pattern Recognition (cvpr) (Los Alamitos: IEEE Computer Soc), 16965–16974. doi: 10.1109/CVPR52733.2024.01605

[B37] Zhao C. Ren Z. Li Y. Zhang J. Shi W. (2025). Growth stages discrimination of multi-cultivar navel oranges using the fusion of near-infrared hyperspectral imaging and machine vision with deep learning. Agriculture-Basel 15, 1530. doi: 10.3390/agriculture15141530 30654563

[B38] Zhou B. Wu K. Chen M. (2024). Detection of Gannan navel orange ripeness in natural environment based on YOLOv5-NMM. Agronomy-Basel 14, 910. doi: 10.3390/agronomy14050910 30654563

